# Religious involvement and racial disparities in opioid use disorder between 2004–2005 and 2012–2013: Results from the National Epidemiologic Survey on Alcohol and Related Conditions

**DOI:** 10.1016/j.drugalcdep.2019.107615

**Published:** 2019-10-17

**Authors:** Yusuf Ransome, Angela M. Haeny, Yoanna E. McDowell, Ayana Jordan

**Affiliations:** a Yale School of Public Health, Department of Social and Behavioral Sciences, Studies of Religion Ethnicity Technology and Contextual Influences on Health (STRETCH)-Lab, 60 College Street, New Haven, CT 06510; b Yale School of Medicine, Department of Psychiatry, Division of Prevention and Community Research, and The Consultation Center 389 Whitney Avenue, New Haven, CT 06511; c University of Missouri, Alcohol, Health, and Behavior Lab, Department of Psychological Sciences, 146 Psychology Building, Columbia, MO 65211; d Yale School of Medicine, Department of Psychiatry, and Connecticut Mental Health Center 40 Temple Street, New Haven, CT 06510

**Keywords:** Opioid Use Disorder, Race/ethnicity, NESARC, Religious involvement, Spirituality

## Abstract

**Background:**

Psychosocial factors have rarely been studied to understand racial differences in opioid use disorders (OUD). We investigate religious involvement and Black-White differences in OUD risk between 2004–05 and 2012–13.

**Methods:**

We use Non-Hispanic Black and White adults from the National Epidemiologic Survey on Alcohol and Related Conditions (wave 2, N = 26,661 and NESARC-III, N = 26,960) (NESARC). We conducted survey-weighted logistic regression to examine whether race moderates the association between religious involvement and lifetime DSM-IV and −5 OUD and whether those differences change (i.e., are modified) by time, adjusted for covariates such as age, education, and urbanicity. Religious involvement measures were service attendance, social interaction, and subjective religiosity/spirituality.

**Results:**

The prevalence of lifetime DSM-IV (3.82 vs 1.66) and DSM-5 (2.49 vs 1.32) OUD in NESARC-III was higher among White compared to Black respondents. Never attending services declined for both races over time. Race moderated the association between service attendance (F(4,65) = 14.9, p = 0.000), social interaction (F(4,65) = 34.4, p = 0.000) and subjective religiosity/spirituality (F(2,65) = 7.03, p = 0.000) on DSM-IV OUD in wave 2 and using DSM-5 OUD in NESARC-III (F(1,113) = 2.79, p = 0.066). Race differences in religion and DSM-IV OUD risk was modified by time (i.e., survey year) (all p < 0.000). For instance, higher service attendance was associated with lower DSM-IV risk for Black respondents in wave 2 but higher risk in NESARC-III. There were no changes in regression slopes among White respondents.

**Conclusions:**

Religious involvement may be important for prevention and treatment practices that respond to racial differences in risk of OUD. Replicate studies should examine other religious factors and specific types of opioids.

## 1. Introduction

Opioid use disorder (OUD) and related deaths is a global public health threat (Martins et al., 2015). Since the start of the 21^st^ century, the rate of opioid overdose deaths has nearly quadrupled, claiming more than 500,000 deaths over the past two decades (Rudd et al., 2016). In 2017 alone, more than 64,000 Americans died from drug overdoses and opioids was the primary cause of death for 47,600 of those cases (Scholl et al., 2019).

There was one clear trend by race/ethnicity in the epidemiology of opioid misuse and disorder from late-1990s to around 2013. On average, Black compared to White people had lower prevalence of non-medical prescription OUD both in the past 12 months and over the lifetime. (Saha et al., 2016; Vaughn et al., 2016) and differences exist even after adjusting for confounders. There is still is no clear explanation for this trend by race/ethnicity (Hasin and Grant, 2015).

Reasons that have been put forth to explain Black-White differences in OUD focused overwhelmingly on health care-related factors. For instance, some note that White compared to Black people have higher access to opioid prescriptions at emergency rooms and higher exposure to opioids per capita population (Friedman et al., 2019). Others note that Black people have been shielded from the brunt of the epidemic due to physician-patient dynamics such as bias and racially discriminatory-based prescribing practices (Burgess et al., 2008; Hoffman et al., 2016; James and Jordan, 2018).

Beyond health care, protective psychosocial factors, specifically, religiosity has been overlooked but is critical to understanding the observed patterns of Black-White differences in the opioid epidemic (Harrell and Broman, 2009; Rigg and Nicholson, 2019). Opioids are highly addictive, so primary prevention is the best intervention. Religiosity has been theorized to reduce one’s risk of becoming physiological and psychologically dependent on substances by lowering the likelihood of initial use—primary prevention, and curtailing continued use—secondary prevention (Gorsuch, 1995; Miller, 1998). Substantial empirical support has shown that higher religiosity and spirituality are often associated with better mental health outcomes, which includes lower substance use disorders, (Idler et al., 2003; Nelson, 2009) and remission from drug dependence (Schoenthaler et al., 2015).

The first hypothesis in this paper is that any protective aspects found between religiosity and OUD risk might function differently by race, in a direction favoring Black people. The positive impact of religiosity on OUD and substances in general is dependent on the level of commitment or engagement in religious activity or with religious people, and the interpretation or receptivity to the religious message (Gorsuch, 1995). We expect lower OUD among Black people because, regardless of ethnicity or nativity (e.g., Caribbean, African-born), they are more religiously engaged than White people (Pew Forum on Religion and Public Life, 2014; Taylor et al., 2014, 1996). Therefore, the effect of any preventive aspects of religiosity, including social support, spiritual coping (Holt et al., 2018) and referrals to treatment (Wong et al., 2018) should be higher among Black people.

Another expectation, based on research from the alcohol use literature (Ransome and Gilman, 2016; Ransome et al., 2019; Szapocznik et al., 2007) is that at equivalent levels of religious engagement between Black and White people, we could expect that Blacks might experience a stronger protective association against OUD. Black may be more likely than White people to reverence social norms, submit to sanctions of delinquent substance use behavior (Krause, 2003; Ransome, 2014) but also positively interpret internally any hope, fear, or condemnation from religious teachings. Several foundational works on religiosity and spirituality among people of the Black and African diaspora support why such a hypothesis might be plausible. These works show that religiosity and spirituality is a central part of the Black human experience and a core part of Black-African people’s social and cultural identity and resistance from oppression (Stewart, 1997; Wilmore, 2004).

Today, the racial/ethnic epidemiology of OUD has been changing. National trends show that both Black and White people are experiencing exponential increases in opioid misuse and mortality (Chicago Urban League et al., 2017; Harrison et al., 2018; James and Jordan, 2018). Some surveys report that non-medical prescription use and misuse rates are similar (Han et al., 2017) or statistically non-different between Black and White adults (Nicholson and Ford, 2018). Others observe that Black-White differences have narrowed and eventually became statistically non-different among adolescents (Vaughn et al., 2016).

Beyond opioid misuse, some studies showed higher opioid-related deaths rates among Black compared to White people, after data are stratified by specific opioid type such as heroin, synthetic opioids, and prescription opioids (Alexander et al., 2018; Scholl et al., 2019); and when stratified by age group and sex (Allen et al., 2019; Shiels et al., 2018).

Health-care related factors such as physician prescribing practices are unlikely to fully explain recent Black-White trends of opioid misuse today, although physicians are beginning to address their biases in prescribing practices to deliver equitable treatment for all (Medlock et al., 2019). We assert that religious involvement again, is critical to understand this narrowing of Black-White differences in OUD today.

Religious involvement is declining in the United States and engagement is significantly lower among millennials—the generation that is currently experiencing the brunt of the epidemic, compared to those born in earlier generations and periods of the epidemic (Pond and Pew Research Center, 2010; Smith et al., 2019). Trend data we analyzed from the General Social Survey shows significant increases between 2005 and 2019, among both Black and White people in the prevalence never attending religious services (Smith et al., 2019). Therefore, if a greater proportion of people are not exposed to religious instruction, norms, supports or other aspects of religious life, they may be more susceptible to opioid use and OUD.

The second hypothesis in this paper is that any race by religiosity differences on OUD will be different and weaker between NESARC wave 2 and NESARC-III.

## 2. Methods

### 2.1. Data

#### 2.1.1. Sample

Data were from the NESARC wave 2 conducted in 2004–2005 (Grant et al., 2009). NESARC is a population-based survey that captured health outcomes, behavioral factors, and psychiatric disorders among civilian non-institutionalized adults in the United States (U.S.) (National Institutes of Health and National Institute on Alcohol Abuse and Alcoholism, 2010). NESARC oversampled non-Hispanic Black and Hispanic persons aged 18–24 years. Extended details of the sampling methodology are published (Grant and Dawson, 2006; Grant et al., 2009). Wave 2 consisted of 34,653 interviews with a response rate of 87 percent. The wave 2 sample was restricted to non-Hispanic Black and White respondents only (*n* = 26,661), hereafter. Additionally, data from the most recent edition, NESARC-III (Grant et al., 2014), were also used to determine whether racial differences in the association between religion and OUD persisted or changed over time. NESARC-III was also restricted to Black and White respondents (*n* = 26,960). Study protocols for our statistical analysis with NESARC wave 2 and NESARC-III were reviewed by Yale University (IRB 2000024424) and was determined not human subjects research.

### 2.2. Measures

#### 2.2.1. Non-medical use of prescription opioids (hereafter, opioids)

OUD was measured with the Alcohol Use Disorder and Associated Disabilities Interview Schedule-IV (AUDADIS-IV)(Grant et al., 2003) according to the DSM, Fourth Edition (DSM-IV) (American Psychiatric Association (APA), 2000) in NESARC wave 2. In NESARC-III, OUD was measured by the AUDADIS-5 (Grant et al., 2011), based on the DSM-5. Non-medical use of prescription opioids in NESARC referred to using prescription medications, in greater amounts, more often, or longer than prescribed, or for a reason other than prescribed by a doctor or physician. Respondents were provided an extensive list of examples of prescription opioids (Demerol, Vicodin, Buprenex, hydrocodone, and oxycodone) and asked if they used any of the prescription opioids on the list or similar drugs ‘non-medically’. If the response was yes, the respondent was asked to specify which prescription opioid they used, and when they used it (lifetime, past year, since last interview/prior-to-past year).

Lifetime diagnoses of abuse required the participant to meet at least one of the four criteria defined for DSM-IV abuse. Lifetime diagnoses of OUD required at least three of the seven DSM-IV criteria for dependence to be met within the same one-year period. OUD abuse and dependence was combined into binary variablebecause of small cell sizes and to be consistent with DSM-5 scoring; 1= yes (met criteria for OUD), 0= no in both samples. An additional lifetime OUD diagnosis variable based on DSM-5 was also created for the NESARC-III sample only. The DSM-5 OUD diagnosis was based on the endorsement of at least two DSM-5 OUD criteria at any time in a twelve-month period, where 1= yes (met criteria for OUD), and 0= no.

#### 2.2.2. Religious involvement

In both NESARC datasets, there are four religious involvement questions in common. *Religious service attendance* was ascertained with two questions: 1) whether respondents currently attend religious services at a church, mosque, synagogue or other religious place. Responses were yes or no. The other question asked about frequency of service attendance. Responses ranged from 1= once a year to 5= twice a week or more. Because frequency of service attendance is only recorded among those who attend services, we derived a new variable by adding another category 0= never attend, 1= once a year/a few times a year (collapsed because of small cell size in once a year), 2= one to three times a month, 3= once a week and 4= twice a week or more. *Social interaction* was ascertained from the question: “how many members of your religious group do you see or talk to socially every two weeks?” The variable was categorized based on the distribution into: 1= ≤8 members, 2 = 9 to 16 members, and 3= ≥17 members. *Subjective religiosity and spirituality* was ascertained from the question: “how important are religious or spiritual beliefs in your daily life?” Responses ranged from 0= not at all important to 3= very important and were reverse coded to match the direction of religious service attendance.

#### 2.2.3. Covariates

We selected covariates based on known associations between religion and health outcomes (Idler et al., 2003; Koenig, 2011; Nelson, 2009) and our own knowledge of the topic (Ransome and Gilman, 2016). Potential covariates for the multivariable analyses were: continuous age, sex (1= men, 0= women), marital status (0=married and cohabitating, 1=widowed, separated, or divorced, and 2=never married), nativity status (0=US-born, 1=foreign-born), educational attainment (0=less than high school, 1=completed high school, 3=college degree, 4=graduate education or higher), personal income (0= ≤$19,999, 1= $20,000 to $34,999, 2= $35,000 to $69,000, and = ≥$70,000), census region (0=northeast, 1=midwest, 2=south, 3=west).

In NESARC-III there is no longer an MSA variable but one that describes urban vs. rural residence. Consistent with prior studies that combined NESARC wave 2 and NESARC-III (Martins et al., 2017), we recoded the MSA variable into 1= urban (i.e., in an MSA either central or not) and 0= rural (i.e., not an MSA) to keep both variables the same. We also included self-rated health (ranged from 0= poor to 4= excellent), past-year health insurance coverage (0= no health insurance coverage, 1= health insurance coverage), and lifetime racial/ethnic discrimination. Lifetime racial/ethnic discrimination was measured by a version of the Experiences of Discrimination scale developed by Krieger and colleagues (Krieger et al., 2005) that was adapted for NESARC (Ruan et al., 2008). There were six items total and each response ranged from 0= never to 4= very often. Items were assessed in the past year and prior-to-the-past year. The six items were summed to create a racial/ethnic discrimination score for each time period.

### 2.3. Statistical analysis

#### 2.3.1. Descriptive

Un-weighted sample sizes are reported with weighted percentages for categorical variables and weighted means and standard errors are reported for continuous variables. Statistical significance in the covariates and OUD prevalence were calculated using Rao-Scott Chi-squared test for categorical variables and Wald-F tests for continuous variables. Those results are presented in Table 1.

#### 2.3.2. Bivariate

Given sex (Maselko and Kubzansky, 2006) and racial differences in religious involvement (Levin et al., 1994), we first explored the interaction between sex and race on OUD. The interaction was not statistically significant, so we proceeded with men and women included in pooled estimates. We use STATA’s 14.0 (StataCorp, 2014) suite of “svy” and “subpop” commands. Analyses using the “svy” procedures account for the complex survey design of NESARC wave 2 and NESARC-III and obtain correct standard errors when analyzing subgroups within a larger survey (Heeringa et al., 2010). Given the small cell sizes among persons with OUD, we recoded religious service attendance into 0= never, 1= once a year to one to three times a month, 2= once a week, and 3= twice a week or more, and subjective religiosity and spirituality into 0= not important/not very important/somewhat important, and 1= very important.

#### 2.3.3. Multivariable

To begin examining race differences, we fit logistic regressions to estimate the lifetime risk of OUD for all religious involvement measures simultaneously but stratified the analysis by NESARC survey year (i.e., wave 2 and NESARC-III). Including all religious involvement items together is important to avoid misspecification (Taylor et al., 1999). Then, we pooled the two NESARC datasets and specified the same equation but then including a three-way interaction for race*survey year*each religion variable. STATA software automatically produces all the relevant two-way interaction terms (e.g., race*survey year, race*religion, etc.,). Then, we tested for effect modification (i.e., significance in race*time differences) using the Adjusted Wald Test through the *contrast* command in STATA 14, which reports degrees of freedom, an F-score, and a *p*-value. We determined interactions statistically significant at the *p* < 0.10 level. The results from this analysis provides an omnibus/overall test statistic for whether the association between religion and race on OUD varies over time.

Next, we conducted stratified analyses by race, this time specifying the two-way interactions between the religion variables and survey year and we used STATA’s *margins* command to retrieve the predicted probabilities of OUD separated by year, which we display in figures. Presenting the data in this format allows one to see the influence of time within race.

All analyses to this point used DSM-IV OUD. To investigate race differences using DSM-5 OUD, we used NESARC-III to fit a regression model to estimate potential effect modification by race on the association between the religion variables on DSM-5 OUD, adjusting for the same covariates in prior steps.

We conducted post-hoc exploratory cross tabulation between race and Census region among people with DSM-IV OUD in NESARC wave 2 and NESARC-III to help us understand our non-significant findings between race and religion on DSM-IV OUD in NESARC-III.

## 3. Results

### 3.1. Descriptive

Overall, Black compared to White respondents were significantly younger (mean age 45.42 vs. 49.90 in NESARC wave 2 and 43.38 vs 48.88 in NESARC-III). Both NESARC survey years were comprised of a higher percentage of Black compared to White women (56.30 vs 51.87 in NESARC wave 2 and 59.11 vs 54.89 in NESARC-III). Significantly fewer proportion of Black compared to White respondents reported never attending religious services in both survey years (31.10 vs 49.00 in NESARC wave 2 and 34.43 vs 54.85 in NESARC-III). The proportion of never attenders in both groups increased from NESARC wave 2 to NESARC-III. Last, the lifetime prevalence of DSM-IV OUD was significantly lower among Black respondents in both survey years (*p* <0.000) and when DSM-5 OUD was assessed in NESARC-III (1.32% vs 2.49%).

### 3.2. Religious involvement, race, and opioid use disorder

We found partial support for the first hypothesis that there are race differences in the association between religious involvement and OUD risk. Race moderated the association between service attendance (F(4,65) = 14.9, p = 0.000), social interaction (F(4,65) = 34.4, p = 0.000) and subjective religiosity/spirituality (F(2,65) = 7.03, p = 0.002) on lifetime risk of OUD in NESARC wave 2 only when DSM-IV was applied, and subjective religiosity/spirituality (F(1,113) = 2.79, p = 0.066) in NESARC-III when DSM-5 criteria was applied (Table 2). Based on our post-hoc analysis, cross-tabulations revealed that in NESARC wave 2, among those with DSM-IV OUD, 45% of Black respondents were from the South and 12% from the Midwest but in NESARC-III 37% of Black respondents were from the South and 32% from the Midwest. In NESARC-III, 38% of White respondents with OUD were from the South and 21% from the Midwest.

In NESARC wave 2, compared to those who seldom attended; attending services twice per month was associated with almost four times higher risk of DSM-IV OUD for White compared to Black respondents (Adjusted Odds Ratio (aOR) = 3.58, 95%CI 2.15, 5.95). Compared to those who rated subjective religiosity/spirituality as very important; the risk of OUD was .45 times lower for White compared to Black respondents who rated it less than very important in NESARC wave 2 using DSM-IV (aOR = 0.45, 95%CI 0.31, 0.66) and about 0.57 times lower in NESARC-III using DSM-5 criteria (aOR = 0.45, 95%CI 0.31,0.66) (Table 3).

**Fig. 1.**
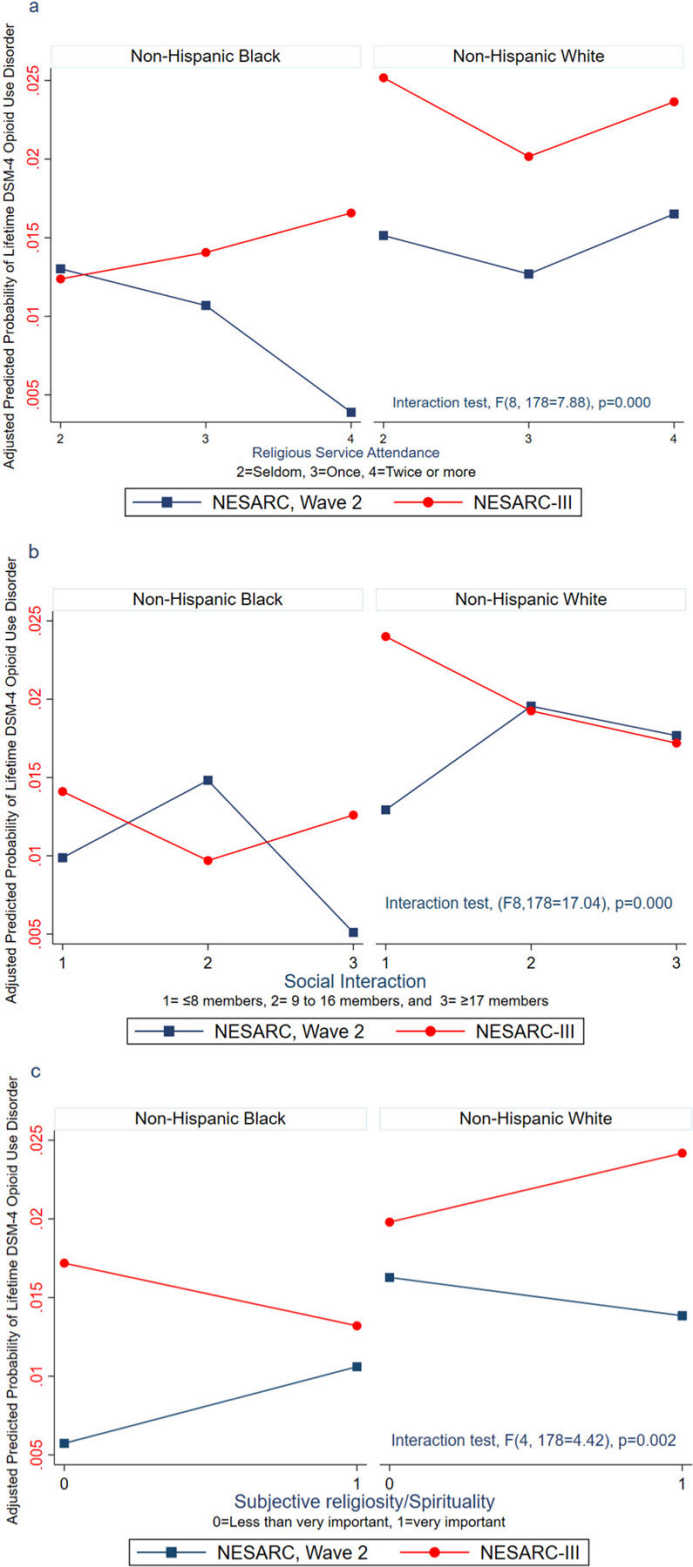
a) Higher service attendance associated with lower risk of OUD in NESARC wave 2 but increasing risk of OUD in NESARC-III for Black but not White respondents. b) Interaction with 17 or more members associated with lower OUD risk in NESARC wave 2 but higher risk in NESARC-III for Black but not White respondents. c) Rating subjective religiosity/spirituality as very important compared to less than important associated with higher OUD risk in NESARC wave 2 but lower OUD in NESARC-III for Black, but the pattern is reversed for White respondents. **Notes:** Figures are based on the regression model adjusted for age, sex, marital status, education, income, health insurance, nativity status, self-rated health, lifetime discrimination, and residence in an urban or rural area. Each religious involvement variable and a two-way interaction with race are included simultaneously in the same regression.

We found stronger support for our second hypothesis that race by religiosity differences on OUD will be different and weaker in NESARC-III compared to NESARC wave 2. Race by religion association with lifetime DSM-IV OUD was significantly different between the NESARC survey years for service attendance (F(8,178) = 7.88, p = 0.000), social interaction (F(8,178) = 17.04, p = 0.000) and subjective religiosity/spirituality (F(4,168) = 4.42, p = 0.002). These differences are presented in Fig. 1a–c, which are plots of the adjusted odds ratios transformed into predicted probabilities.

Fig. 1a shows that, among Black respondents, the predicted risk of DSM-IV OUD declined with increasing frequency of service attendance in NESARC wave 2 but increased in NESARC-III. Among White respondents, the intercept only was lower in NESARC wave 2 and higher in NESARC-III, but the associations (i.e., slopes) with increasing attendance were parallel.

Fig. 1b shows that, among Black respondents, an increase in social interaction from nine toward 17 or more people was associated with a lower DSM-IV OUD risk in NESARC wave 2 and a higher risk in NESARC-III. Among White respondents, the intercept and the slope remained the same across both NESARC survey years, and there appears to be a flat trend as social interaction increases from nine toward 17 or more people.

Fig. 1c shows that, among Black respondents, the risk of DSM-IV OUD increases as they rate subjective religiosity/spirituality very important compared to less than important, in NESARC wave 2, and the risk decreased for the same rating in NESARC-III. Among White respondents in NESARC wave 2, the risk of DSM-IV OUD was unchanged across ratings of subjective religiosity/spirituality and increased in NESARC-III.

## 4. Discussion

In this study, we investigated whether religious involvement could help illuminate prior and recent epidemiologic trends of Black-White differences in prevalence of lifetime OUD, using two nationally representative NESARC surveys: (wave 2, 2004–05) and (NESARC-III, 2012–13). We examined three religious involvement variables available across both surveys: frequency of religious service attendance, religious social interaction, and subjective religiosity/spirituality. We also used both DSM-IV and DSM-5 criteria.

Our study significantly adds to the literature through five aspects: 1) a focus on adults, 2) examination of multiple religious/spiritual involvement variables, 3) specifically test the effect modification by race, 4) test the effects of race differences over time, and (5) use DSM-IV as well as DSM-5 criteria. Previous studies that included race and religious involvement with OUD were among adolescents or young adults (Harrell and Broman, 2009; Snipes et al., 2015) and (Berenson and Rahman, 2011; Palamar et al., 2014) have not focused explicitly on race differences.

First, we find that Black respondents had a lower lifetime prevalence of OUD risk across both NESARC years when DSM-IV was applied. Although when OUD was assessed with DSM-5, Black respondents still had lower lifetime prevalence, but Black-White differences were narrower than when assessed with DSM-IV.

Next, we find partial but robust support for our two hypotheses across the three religion variables that (1) associations with lifetime OUD risk varies between Black and White respondents and (2) that those associations are diminished between NESARC wave 2 and NESARC-III. Race-differences were found for all religious variables in wave 2 and only among subjective religiosity/spirituality in NESARC-III. These findings highlight the importance of assessing multiple religious involvement indicators because they may be differentially associated with OUD risk.

Specifically, we find that increases in the frequency of attending religious services and interacting with a greater number of members had a stronger and positive impact on lower DSM-IV OUD risk for Black but not for White respondents, in wave 2. These findings that the impact (size or direction) of association varies between Black and White people have been found for alcohol use disorder (Ransome and Gilman, 2016). These findings potentially fit with one theoretical proposition we stated earlier that Black people may be more likely than White people to respond to social norms and sanctions against substance use from religious teachings. Further qualitative evidence is needed to support these statements. We were surprised that in wave 2, race-differences in subjective religiosity/spirituality on OUD was inconsistent with the directions we saw from the others. These findings could be related to reverse causation where Black respondents who are grappling with OUD seek to find meaning in their situation through increasing reliance of religious and spiritual coping (Brome et al., 2000).

By NESARC-III, however, race-differences in the association between the religious variables and DSM-IV OUD were gone. We suspect that the loss of protective status (for attendance and social interaction) could be due to sharper increase in the number of both Black and White respondents who never attended religious services between 2004 and 2014 (Smith et al., 2019). For religion to have an impact, people need to be exposed to religiosity through both messages and interacting with religious people (Gorsuch, 1995). Another possibility is that in NESARC-III, more Black respondents with OUD were from the Midwest, and several states such as Wisconsin, Illinois, and Minnesota have higher rates of opioid overdose deaths per 100,000 for Black people (James and Jordan, 2018).

In NESARC-III, rating spirituality as very important was associated with lower DSM-IV OUD risk and no differences in DSM-5 OUD risk for Black but increasing risk for White respondents regardless of which DSM criteria were applied. We cannot explain these findings but prior work has showed mixed associations between subjective religiosity/spirituality and prescription drug abuse in a national sample of Black adolescents (Harrell and Broman, 2009).

Our study has some limitations. Foremost, while we used two NESARC survey years, these are both cross-sectional data among different individuals. Therefore, we cannot rule out potential reverse causation or other secular changes in the population. We investigated *licit* opioids, which was operationalized as prescription opioids used for non-medical purposes. In NESARC there are *illicit opioids* such as heroin. We acknowledge different racial patterns across types of opioids (Alexander et al., 2018), which we will examine in future work.

Religiosity is a multidimensional construct and was likely not entirely captured by the three indicators here. Future work should include other indicators such as frequency of prayer and employ qualitative and other research methods (Burlew, 2019). Religious denomination was not considered because only NESARC-III contained this variable. Future work should investigate denomination and OUD associations. Despite those limitations, our focus on religiosity and spirituality has important implications for OUD management and prevention, especially among minorities who may be from low-resource settings or delay seeking care because of medical mistrust and racism (Beraldo et al., 2019). Others have already written about the importance of religion and spirituality for Black people dealing with AUD and other substance use and mental disorders (Bohnert et al., 2010; Brome et al., 2000; Taylor et al., 2000). Next, a recent study found that spirituality and religiosity interventions compared to others, were statistically significantly and more effective in helping people recover from substance use problems (Hai et al., 2019).

We recommend that clinicians explicitly incorporate religiosity and spirituality in OUD treatment (Matteliano et al., 2014). The practice is not new (Heinz et al., 2007; Miller, 1998) and others have provided guidelines on how to do this in clinical settings (Christensen et al., 2018; Koenig, 2000). Future work should examine religious and spiritual aspects for prevention of OUD among at-risk populations.

## Figures and Tables

**Table 1:** Distribution of individual-level religious involvement, socio-demographic and health among adults responding to lifetime opioid use disorder question: The National Survey of Alcohol and Related Conditions (NESARC), Wave 2 and NESARC-III.

	NESARC Wave 2			NESARC-III		
	Non-Hispanic Black (n = 6575)	Non-Hispanic White (n = 20,086)	*p*-value	Non-Hispanic Black (n = 7766)	Non-Hispanic White (n = 19,194)	*p*-value
**Service attendance**, n (%)						
Never	1897 (31.10)	10071 (49.00)	0.000	2675 (34.43)	10557 (54.85)	0.000
Once a year to few times a year	640 (09.90)	1421 (07.02)		858 (11.04)	1390 (07.26)	
One to three times a month	1298 (19.34)	2547 (12.91)		1568 (20.14)	2219 (11.60)	
Once a week	1498 (21.91)	4408 (22.17)		1587 (20.48)	3531 (18.49)	
Twice a week or more	1252 (17.74)	1700 (08.89)		1075 (13.91)	1492 (07.79)	
**Social interaction**, n (%)						
< = 8	3585 (76.90)	7286 (71.81)	0.000	4405 (87.21)	6833 (79.72)	0.000
9 to 16	627 (13.66)	1499 (15.56)		417 (08.03)	1137 (13.35)	
> = 17	451 (09.44)	1206 (12.63)		224 (04.48)	590 (06.93)	
**Subjective religiosity and spirituality**, n (%)			0.000			0.000
Not important at all	77 (01.32)	1155 (05.48)		151 (01.98)	1807 (09.39)	
Not very important	123 (02.32)	1935 (09.64)		193 (02.50)	1966 (10.26)	
Somewhat important	1031 (16.88)	6379 (32.04)		1301 (16.86)	5918 (30.97)	
Very important	53333 (79.47)	10603 (52.83)		6109 (78.67)	9472 (49.38)	
**Age**, M (SE)	45.42 (00.11)	49.90 (00.08)	0.000	43.38 (00.32)	48.88 (00.23)	0.000
**Sex**, n (%)						
Men	2326 (43.70)	8853 (48.13)	0.000	3153 (40.89)	8555 (45.11)	0.000
Women	4261 (56.30)	11308 (51.87)		4613 (59.11)	10639 (54.89)	
**Marital Status**, n (%)						
Married/cohabiting	2376 (42.84)	11756 (66.51)	0.000	2158 (28.37)	9752 (52.28)	
Widowed/separated/divorced	2240 (25.43)	5310 (18.96)		2188 (27.87)	5440 (27.24)	
Never married	1971 (31.73)	3095 (14.54)		3420 (43.76)	4002 (20.47)	
**Nativity, Born inside the US**, n (%)	6083 (90.24)	19244 (95.64)	0.002	7189 (92.46)	18265 (95.22)	0.000
**Education**, n (%)			0.000			0.000
Less than high school	1258 (17.61)	2058 (10.06)		1321 (16.96)	1726 (08.95)	
Completed high school	3451 (54.22)	9928 (50.08)		4421 (56.87)	9113 (47.43)	
College degree	1523 (23.43)	6078 (30.00)		1665 (21.51)	6240 (32.49)	
Graduate education and higher	355 (04.74)	2097 (14.61)		359 (04.66)	2115 (11.12)	
**Personal income**, n (%)			0.000			0.000
≤$19,999	3237 (47.69)	8013 (39.52)		4121 (53.02)	7793 (40.56)	
$20,000–$34,999	1644 (25.99)	4578 (22.48)		1886 (24.19)	4096 (21.15)	
$35,000–$69,999	1410 (21.78)	5214 (25.92)		1367 (17.67)	4772 (24.84)	
≥ $70,000	296 (04.55)	2356 (12.09)		392 (05.13)	2533 (13.45)	
**Census Region,** n (%)			0.727			0.680
Northeast	1280 (18.74)	3670 (18.39)		944 (12.44)	2956 (15.95)	
Midwest	1187 (17.85)	3580 (17.63)		1588 (20.26)	5051 (26.27)	
South	2462 (38.43)	7835 (38.70)		4715 (60.46)	6921 (35.91)	
West	1658 (24.98)	5076 (25.28)		519 (06.84)	4266 (21.86)	
**Metropolitan statistical area (MSA) residence**, n (%)			0.903			0.000
Urban	5533 (83.77)	16878 (83.64)		6941 (89.29)	14375 (75.52)	
Rural	1054 (16.23)	3283 (16.36)		825 (10.71)	4819 (25.48)	
**Health insurance coverage,** n (%)			0.000			0.000
Yes	5668 (83.96)	18525 (97.76)		5858 (75.55)	16624 (86.75)	
No	919 (16.04)	1636 (08.24)		1908 (24.45)	2570 (13.25)	
**Lifetime racial discrimination,** M (SE) range (0 = none, 30 = high)	07.88 (0.02)	06.34 (0.00)	0.000	08.86 (0.12)	06.83 (0.03)	0.000
**Self-rated health,** M (SE) range (1=worse, 5 = best)	3.41 (00.07)	3.69 (00.05)	0.000	3.37 (00.03)	3.57 (00.02)	0.000
**Lifetime prevalence of DSM-IV opioid use disorder,** yes, n (%)	69 (1.14)	429 (2.20)	0.000	129 (01.66)	735 (03.82)	0.000
**Lifetime prevalence of DSM-5 opioid use disorder,** yes, n (%)	N/A	N/A		102 (01.32)	480 (02.49)	0.000

*Note.* Column percent is weighted. n = sample size. M = mean, SE = standard error, NA = not available since DSM-5 was not used in NESARC wave 2.

**Table 2:** Race differences between religious involvement and lifetime DSM-4 opioid use disorder among adults are present in NESARC Wave 2 but no longer present in NESARC-III.

	NESARC Wave 2 (n = 14,642)	NESARC-III (n = 13,595)	*p*-value, for Black-White differences comparing wave 2 to NESARC-III
	**Adjusted Odds Ratio (95 % CI)**		
**Service attendance**, n (%)			F (df8, 178) = 7.88 *p* = 0.000
Seldom^a^	1	1	
Once a week	1.03 (0.68, 1.57)	1.16 (0.61, 2.21)	
Twice a week or more	**3.58 (2.15, 5.95)**	1.38 (0.70, 2.72)	
	*p* (df4, 65) 14.92 = 0.000	*p* (df4, 113) 0.61 = 0.660	
**Social interaction**, n (%)			F (df8, 178) = 17.04 *p* = 0.000
< =8	1	1	
9–16	0.87 (0.60, 1.25)	1.22 (0.37, 4.00)	
> = 17	**2.62 (1.78, 3.86)**	0.82 (0.22, 3.10)	
	*p* (df4, 65) 34.37 = 0.000	*p* (df4, 113) 0.71 = 0.586	
**Subjective religiosity and spirituality**, n (%)			F (df4, 168) = 4.42 *p* = 0.002
Very important	1	1	
^b^Less than very important	**0.45 (0.31, 0.66)**	1.58 (0.66, 3.75)	
	*p* (df2, 65) 7.03 = 0.002	*p* (df2, 113) 0.55 = 0.577	

*Note.* Black race is the reference group in these analyses, so coefficients are describing the association/odds for Whites on OUD compared to the odds for Blacks.

a Seldom is defined as once a -year to one to three times a month. n = sample size. 95% CI = 95% confidence interval. F-tests are from a pooled logistic regression model with race X religion variables interaction terms.

b The less than very important is defined as not at all important, not very important, and somewhat important. Model adjusted for age, sex, marital status, education, income, health insurance, nativity status, self-rated health, lifetime discrimination, and residence in an urban or rural area. Each religious involvement variable interaction with race are included simultaneously in the same regression. The p-values below the religion variable coefficients are the official statistical test results from the race X religion effect modification within each survey year.

**Table 3:** Race differences are present between subjective religiosity/spirituality and lifetime DSM-5 OUD, among adults in NESARC-III.

	Non-Hispanic Black (n = 5,043)	Non-Hispanic White (n = 8,552)	*p*-value, for Black-White differences
	**Adjusted Odds Ratio (95 % CI)**		
**Service attendance**, n (%)			F (df4, 113) = 1.02 *p* = 0.399
Seldom^a^	1	1	
Once a week	1.05 (0.58, 1.89)	0.73 (0.51, 1.04)	
Twice a week or more	1.55 (0.80, 3.00)	0.78 (0.48, 1.27)	
**Social interaction**, n (%)			F (df2, 113) = 1.48 *p* = 0.231
< = 8	1	1	
> = 9	0.42 (0.11, 1.66)	0.89 (0.56, 1.40)	
**Subjective religiosity and spirituality**, n (%)			F (df2, 113) = 2.79 *p* = 0.066
Very important	1	1	
^b^Less than very important	0.96 (0.40, 2.27)	**0.57 (0.33, 0.98)**	

Notes. Due to small sample size, the > = 17 category for social interaction was collapsed into two (9–16) to create one group > = 9.

Model adjusted for age, sex, marital status, education, income, health insurance, nativity status, self-rated health, lifetime discrimination, and residence in an urban or rural area.
